# Effect of unintended pregnancy on skilled antenatal care uptake in Bangladesh: analysis of national survey data

**DOI:** 10.1186/s13690-020-00468-1

**Published:** 2020-09-16

**Authors:** Md Nuruzzaman Khan, Melissa L. Harris, Christopher Oldmeadow, Deborah Loxton

**Affiliations:** 1 Department of Population Sciences, Jatiya Kabi Kazi Nazrul Islam University, Mymensingh, Bangladesh; 2 Priority Research Centre for Generational Health and Ageing, Faculty of Health and Medicine, School of Medicine and Public Health, University of Newcastle, Newcastle, Australia; 3 Faculty of Health and Medicine, School of Medicine and Public Health, University of Newcastle, Newcastle, Australia

**Keywords:** Unintended pregnancy, Antenatal care uptake, Informative Bayesian approach, Multilevel logistic regression model, Bangladesh

## Abstract

**Background:**

Around 48% of all pregnancies in low- and middle-income countries are unintended. Unintended pregnancy may contribute to lower use of antenatal care (ANC); however, current research in the area is largely inconclusive due to the methodological approaches applied.

**Methods:**

Responses from 4493 women extracted from the 2014 Bangladesh Demographic and Health Survey (BDHS) were used to assess the association between unintended pregnancy and subsequent uptake of at least one and at least four skilled ANC visits. For this, Bayesian multilevel logistic regression models with informative priors (representing a range of values within which the researcher is certain the true effect of the parameters included lies) were used, adjusting for other factors that affect ANC uptake. Informative priors were selected from the BDHS data collected in 2004, 2007, and 2011.

**Results:**

Around 64% of women in Bangladesh who had at least one pregnancy within 3 years prior to the survey (that ended in a live birth) received ANC at least once, and of these around 32% used ANC at least four times. Mistimed (aOR, 0.73, 95% Cred I, 0.66–0.81) and unwanted (aOR, 0.69, 95% Cred I, 0.64–0.75) pregnancy were associated with reduced odds of attending the recommended minimum of four skilled ANC visits compared with wanted pregnancy. These likelihoods were even lower for at least one skilled ANC visit among women with a mistimed (aOR, 0.59, 95% Cred I, 0.53–0.65) or an unwanted pregnancy (aOR, 0.67, 95% Cred I, 0.61–0.74) than women with a wanted pregnancy.

**Conclusions:**

In Bangladesh, more than one-quarter of women who report an unintended pregnancy at conception and do not terminate the pregnancy are at high risk of not using ANC. It is important for policies to include women with unintended pregnancy in mainstream healthcare services. This will increase the use of ANC and reduce associated adverse consequences.

## Background

Increasing uptake of antenatal care (ANC) has been a long-term focus in low- and middle-income countries (LMICs) to improve maternal health and reduce maternal and child deaths [1]. This special interest is demonstrated by the high priority given to increasing ANC uptake in programs run by LMIC governments (e.g. Community-Based Health Planning and Services Programs in Ghana, Maternal and Child Health Care Programs in Bangladesh) and their collaborative programs with donor agencies (e.g. Sahel Women’s Empowerment and Demographic Dividend, 2014, Global Financing Facility, 2015) [2–4]. Additionally, the Millennium Development Goals (MDGs) between 2000 and 2015 had the key priority of increasing ANC uptake [2]. Accordingly, substantial progress has been made in the uptake of ANC, particularly in the MDG period (increased from only 1 in 4 women in 1990 to nearly 1 in 2 women in 2015) [5]. However, this is insufficient, as more than half of women living in LMICs do not receive any ANC during pregnancy, with significant within-country variations [6–10]. Consequently, around 303,000 maternal deaths, 2.6 million stillbirths, and 2.7 million newborn deaths occur in LMICs because of preventable causes related to pregnancy and childbirth [11]. Therefore, strengthening programs to increase ANC uptake is being prioritised in the coming decades and has been added as a target for LMICs to achieve Sustainable Development Goal 3 (SDG 3, 2015–2030), which focuses on reducing preventable maternal and under-five deaths [12].

Bangladesh has achieved remarkable progress in increasing ANC uptake following the implementation of the MDGs (27% in 1993 to 64% in 2014, of which 32% received ANC at least four times), which could have contributed to a substantial reduction in maternal deaths (181 per 100,000 live births in 2014) and under-five deaths (36 per 1000 live births in 2014) [13, 14]. However, it remains a daunting challenge to further reduce maternal and under-five deaths and increase uptake of ANC (part of universal health coverage), which are targeted in the SDGs. Multiple factors have been hindering this process, indicating that a careful analysis of healthcare service use is warranted, along with additional comprehensive interventions around ANC uptake in Bangladesh as well as other LMICs [15]. So far, research has suggested that reductions of educational and wealth gradients in ANC uptake and ensuring an equal level of ANC utilization in rural and urban areas are major challenges [16–19]. Other frequently reported challenges to increasing ANC utilization are as follows: high parity of women, lower autonomy to visit healthcare centres, lower exposure to healthcare services messages and consequent less knowledge regarding the importance of ANC uptake, and unemployment or being engaged in a marginal job [16, 17, 20, 21].

Around half of all pregnancies in LMICs, including Bangladesh, are unintended [22]. Of these, 55% are ended by abortion, which results in one maternal death every 8 min and 5 million women suffering long-term health complications [22, 23]. Complications during and following delivery are also common among women having continued unintended pregnancies and is a major cause of maternal [22, 23], neonatal, and infant deaths in LMICs [24–26]. A global estimate by the Guttmacher Institute found that around a 92% reduction in these deaths could be made by ensuring maternal healthcare services utilization (including ANC uptake) [27]. This indicates a need to explore the pattern of ANC use among women who have an unintended pregnancy and could be considered as critical to the speedy achievement of SDG 3 in LMICs and Bangladesh. However, studies with a major focus on this crucial subject are limited and findings are inconclusive. For instance, an analysis found that women in 32 LMICs who had an unintended pregnancy used ANC around 24% less than women with a wanted pregnancy [21]. Subgroup analysis of these women across regions of countries found similar negative effects for sub-Saharan Africa and Latin America, while no effect was reported for Asia [21]. Conversely, country-level research in sub-Saharan Africa found highly inconsistent results with no [6, 7, 28] or negative effects of unintended pregnancy on ANC uptake [29–31]. Studies in Asian countries, including India and Bangladesh, found negative effects of unintended pregnancy on adequate (at least four visits) ANC [24, 32]. Meanwhile, a recent study in Bangladesh found no evidence of an association between unintended pregnancy and ANC uptake and reported that socio-demographic determinants and socio-economic inequality were major contributors to inadequate ANC uptake [8]. There are several factors that might lead to such disagreements, including different healthcare policies across countries and use of single-level analysis techniques with a limited number of confounders [21, 24, 28, 33].

One of the main reasons for such differing results is related to the methodological approaches applied. All studies were conducted to test the hypothesized association between unintended pregnancy and ANC uptake; however, they ignored the available knowledge (i.e. the sort of association found in previous research) regarding this association. Importantly, incorporating this knowledge in the model would help researchers run more stable models to identify the nature of the true relationship. Moreover, the previously conducted research, particularly in Bangladesh, considered a limited number of confounders and ignored the hierarchy of the data, such as individuals nested within households and households nested within communities. The Bayesian approach with multilevel modelling allows researchers to incorporate available knowledge in the model, along with considering the hierarchy of the data.

In order to determine whether unintended pregnancy is a true challenge to ANC uptake, it is important to identify the nature and strength of any association between pregnancy intention and services use. Consequently, this study was conducted to assess the association between unintended pregnancy and ANC uptake after adjusting for other factors that affect ANC uptake through the Bayesian multilevel logistic regression models with informative priors.

## Methods

### Study overview

The dataset used for this study was the nationally representative cross-sectional 2014 Bangladesh Demographic and Health Survey (BDHS). The National Institute of Population Research and Training (NIPROT) in Bangladesh conducted this survey as part of the Demographic and Health Survey (DHS) program. Financial and technical support was provided by the United States Agency for International Development (USAID). The Bangladesh government and the DHS institutional review board provided scientific clearance and authorization to conduct this survey. Data were collected by using two-stage cluster sampling. A total of 600 enumeration areas (clusters) were selected in the first stage from the National Population and Housing Census sampling frame, with the probability of selection proportional to the unit size [13]. At the second stage, 30 households were selected within each primary sampling unit with systematic random sampling applied. Further details about sampling and other related issues of the BDHS can be found elsewhere [13].

### Sample

We analysed data from 4493 women selected from all 600 clusters included in BDHS, 2014. The criteria used for inclusion in this study were i) gave birth at least once within 3 years preceding the survey and ii) responded to questions on pregnancy intention and ANC visits.

### Outcome variables

ANC uptake was the outcome variable, categorized as at least one skilled ANC visit and at least four skilled ANC visits (WHO’s recommendation at the time of the survey) [34, 35]. Each surveyed woman who had given birth within 3 years preceding the date of interview was asked about whether she received any ANC during her pregnancy. Women who responded positively to this item were asked about the sources and number of times they received ANC. Responses were then categorized as follows: i) uptake of at least one skilled ANC visit (‘0’ if she had no ANC visit from skilled health personnel, ‘1’ otherwise); and ii) uptake of at least four skilled ANC visits (‘0’ if she had fewer than four ANC visits from skilled health personnel, ‘1’ otherwise). We considered qualified doctors, nurses/midwives/paramedics, family welfare visitors, community skilled birth attendants, and sub-assistant community medical officers as skilled health personnel, following the classification used in BDHS 2014 [13].

### Exposure variables

The *main exposure* variable was pregnancy intention based on a woman’s feelings at the time of conception about her most recent pregnancy which ended in a live birth. Pregnancy intention was categorized as either wanted (a pregnancy that was planned and desired), mistimed (a pregnancy that was wanted but occurred earlier than desired), or unwanted (a pregnancy that occurred when no children were desired).

Exposure variables that were considered as confounders of the association between pregnancy intention and ANC uptake were first identified by reviewing previous relevant literature published about ANC uptake in LMICs and Bangladesh [6–9, 21, 28, 30, 31, 36]. The variables identified were then tested for their level of associations with at least one skilled ANC visit and at least four skilled ANC visits. Variables found to be significant (*p = 0.20*) were then summarized using the socio-ecological model of health in three hierarchical levels: individual level, household/family level, and community level [37]. At the *individual level,* women’s age at delivery (categorised as ≤19 years, 20–34 years, and ≥ 35 years), women’s educational status (illiterate, primary, secondary, and higher), and quality of available ANC (treated as a continuous variable) were included. Total number of children ever born (≤2 and > 2), husband’s education (illiterate, primary, secondary, and higher), husband’s occupation (agricultural worker, physical labourer, service worker, business person, and other), index of exposure to mass media (non-exposed, moderately exposed, and highly exposed), and household wealth index (poorest, poorer, middle, richer, and richest) were included as *household/family-level* factors. Women’s response to the questions related to the frequency of reading newspapers, listening to the radio, and watching television in a week were used to generate the index of exposure to mass media, following previous studies in Bangladesh [38, 39]. Women were classified as having no exposure to mass media if they did not access any of the three media at least once a week; as having moderate exposure to mass media if they accessed any of these media at least once a week, and as having high exposure to mass media if they accessed all three media at least once a week. *Community-level factors* that were considered in the analysis were the place of residence (urban and rural), region of residence (Barishal, Chattogram, Dhaka, Khulna, Rajshahi, Rangpur, and Sylhet), community-level literacy (≤25%, 26–50, and > 50%), community-level poverty (high, moderate, low, and middle-to-richest), and community-level uptake of at least four ANC visits (> 50% and ≤ 50%). These variables were categorized following previous studies in Bangladesh [40, 41]. Women’s responses to the questions related to literacy, poverty, and ANC use across clusters were used to generate community-level responses on literacy, poverty, and ANC use, which were not directly available in the dataset used.

### Statistical analysis

We summarised the demographic profile of the eligible women of the 2014 study using means and standard deviations. The proportion of women with at least one skilled ANC visit, the proportion of women with at least four skilled ANC visits, and the proportion of each pregnancy intention (wanted, mistimed, and unwanted) were reported with 95% confidence intervals. Chi-square tests were used to assess the significance of the exposure variables with at least one skilled ANC visit and at least four skilled ANC visits. Multilevel logistic regression models with informative Bayesian approach were then used to assess the association between pregnancy intention (wanted, mistimed, and unwanted) and uptake of i) at least one skilled ANC visit and ii) at least four skilled ANC visits. The advantages of using this methodology have been described in detail elsewhere [42, 43]. Briefly, the model estimates the posterior probability distribution of parameters (for example, the odds ratio for the effect of pregnancy intention on at least one skilled ANC visit) by combining informative prior information (in the form of a distribution representing a range of plausible values) with the likelihood of the current data. Informative prior distribution of the regression coefficients were constructed by first fitting separate Bayesian multilevel logistic regression models with non-informative priors (mean = 0, variance = 10,000) to each of the 2004, 2007 and 2011 BDHSs. We then modelled this prior distribution as independent normal distributions (one for each coefficient) in the proposed model, with means equal to the average of the previous year’s regression coefficient and variance equal to the square of the regression coefficient standard deviation. Multilevel mixed-effects logistic regression models were used to estimate the maximum likelihood estimators. These were then used to estimate posterior distribution using the Markov Chain Monte Carlo (MCMC) method. We ran three different models separately for the uptake of at least one skilled ANC visit and uptake of at least four skilled ANC visits. Model 1 incorporated individual- and household-level factors, while Model 2 incorporated only community-level factors to present the different effects of individual-, household-, and community-level factors on uptake of at least one skilled ANC visit and uptake of at least four skilled ANC visits. We considered individual- and household-level factors together because of their strong correlation and because the average number of women per household in the BDHS was small, so household measures could not be analysed separately. Model 3 was the final model and included individual-, household-, and community-level factors. An additional model run was a null model, estimating intra-class correlation (ICC), which measured overall variation in least one skilled ANC visit and uptake of at least four skilled ANC visits. We used the Gelman-Rubin convergence diagnostic test to check the convergence of the MCMC method, which fits well and provided evidence of the accuracy of the analysis. All analyses were conducted taking into consideration the complex structure of the BDHS data and the survey weight. The posterior distributions are summarised and reported as odds ratios (ORs) with 95% credible intervals (95% Cred.I). Stata version 15.1 (Stata Corp, College Station, Texas, USA) was used to perform all statistical analyses.

## Results

A total of 17,863 women completed the 2014 BDHS (the most recent survey), of which 4493 gave birth within 3 years prior to the survey and responses to the questions on ANC uptake and pregnancy intention were included in the analysis. Table 1 shows the background characteristics of the women. The mean age at most recent delivery was 23.6 (±5.7) years and the mean years of education was 6.3 (±3.8) years. Around one-quarter of all pregnancies that occurred within the preceding 3 years of the survey were unintended (26.0%), of which 15.1% were reported as mistimed and 10.9% were reported as unwanted. Around 64% of women reported having at least one skilled ANC visit during their last pregnancy, of which 31.5% reported uptake of the recommended minimum four skilled ANC visits.

**Table 1:** Background characteristics of the study population, BDHS 2014 (weighted sample *N* = 4493)

Characteristics	Subject (N)	Estimates (mean ± SD or proportion (95% CI))
Age at most recent birth given, years	4493	23.6 (5.7)
Education, years	4493	6.3 (3.8)
Total number of children	4493	2.16 (1.4)
Engagement in formal employment	4493	23.9 (21.6–25.9)
Pregnancy intention (most recent birth)		
Wanted	3362	74.1 (71.3–76.7)
*Unintended*		26.0 (23.3–28.8)
Mistimed	670	15.1 (13.4–16.8)
Unwanted	461	10.9 (9.4–12.6)
Antenatal care services visit		
At least one visit	2927	63.8 (60.4–67.1)
Four or more visits	1188	31.5 (28.8–34.5)

**Fig. 1:**
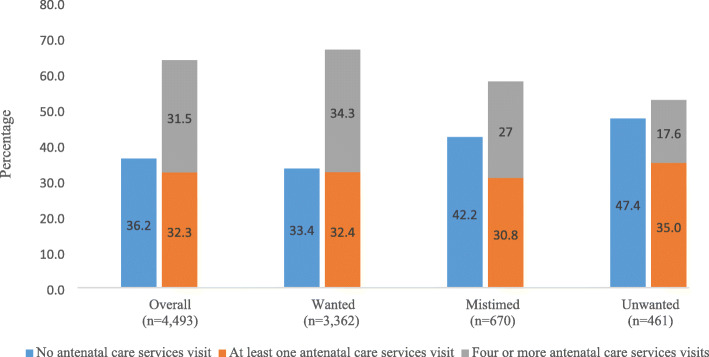
At least one and four or more antenatal care uptake in Bangladesh across the intention of women’s last pregnancy based on BDHS, 2014 data

Figure 1 shows the proportion of women who took up at least one and four skilled ANC visits according to the pregnancy intention regarding their last child. Around 67% of women who had a wanted pregnancy reported attending at least one skilled ANC visit. This was higher than women who had a mistimed pregnancy (57.8%) and women who had an unwanted pregnancy (52.6%). Furthermore, a higher proportion of women who had a wanted pregnancy (34.3%) met the four or more ANC visit target, followed by women who had a mistimed pregnancy (27%) and women who had an unwanted pregnancy (17.6%).

Uptake of at least one skilled ANC visit and at least four skilled ANC visits across selected individual-, household-, and community-level factors are presented in Table 2. Both at least one skilled ANC visit and at least four skilled ANC visits were found to be higher among women aged 20–34 years at the time of giving birth, women not engaged in any jobs, women of low socio-economic status, and women residing in Dhaka division. Primary and secondary educated women reported a higher uptake of at least one skilled ANC visit compared to higher educated women. In contrast, secondary educated women reported a higher uptake of at least four skilled ANC visits.

**Table 2:** Uptake of at least one skilled antenatal care visit and at least four skilled antenatal care visits, by selected characteristics, 2014 BDHS (weighted sample *N* = 4493)

Characteristics	Prevalence (95% confidence interval)			
	At least one skilled antenatal care (***n*** = 2927)	***p-value***	Four and more skilled antenatal care (***n*** = 1188)	***p-value***
**Pregnancy intention**		*p < 0.01*		*p < 0.01*
Wanted	49.3 (45.6–52.8)		25.9 (23.0–19.0)	
Mistimed	8.7 (7.8–9.7)		3.9 (3.2–4.8)	
Unwanted	5.8 (5.0–6.7)		1.8 (1.1–2.7)	
**Women education**				
No education	5.5 (4.7–6.5)	*p < 0.01*	1.6 (1.0–2.6)	*p < 0.01*
Primary	14.4 (13.2–15.8)		4.9 (4.0–5.9)	
Secondary	3.5 (31.9–37.4)		17.8 (15.7–20.1)	
Higher	9.2 (8.1–10.6)		7.3 (6.2–8.5)	
**Women age birth (years)**		*p < 0.05*		*p < 0.05*
≤ 19	17.9 (16.1–19.8)		8.3 (7.0–9.8)	
20–34	43.3 (40.8–45.8)		22.2 (20.1–24.5)	
≥ 35	2.6 (2.0–3.2)		1.1 (0.7–1.5)	
**Women’s working status**		0.783		0.215
Yes	49.7 (43.7–55.8)		28.1 (25.5–30.9)	
No	50.6 (47.4–53.7)		24.3 (19.2–30.2)	
**Husband educational status**		*p < 0.01*		
No education	10.2 (9.1–11.4)		3.0 (2.4–3.9)	*p < 0.01*
Primary education	16.7 (14.9–18.7)		6.5 (5.3–7.8)	
Secondary education	23.9 (21.9–26.1)		12.5 (10.1–14.4)	
Higher education	12.9 (11.4–14.5)		9.6 (8.2–11.1)	
**Husband occupation**		*p < 0.01*		
Agricultural workers	12.2 (10.6–13.9)		4.4 (3.4–5.5)	*p < 0.01*
Physical labourer	28.9 (26.4–31.1)		14.2 (12.7–15.9)	
Service worker	5.2 (4.3–6.2)		4.2 (3.3–5.2)	
Business person	15.8 (14.4–17.3)		7.7 (6.6–9.0)	
Others	2.0 (1.4–2.9)		1.1 (0.5–2.1)	
**Children ever born**				
≤ 2	47.9 (44.5–51.3)	*p < 0.01*	25.6 (22.9–28.6)	*p < 0.01*
> 2	15.9 (14.6–17.2)		5.9 (5.0–7.0)	
**Exposure to mass media**				
Not exposed	17.5 (15.8–19.3)	*p < 0.01*	5.0 (4.1–6.2)	*p < 0.01*
Moderately exposed	34.3 (31.8–36.8)		17.6 (15.8–19.4)	
Highly exposed	12.0 (10.5–13.6)		9.0 (7.6–10.5)	
**Wealth status**				
Poorest	7.7 (6.6–8.9)	*p < 0.01*	1.9 (1.5–2.6)	*p < 0.01*
Poorer	10.6 (9.4–11.8)		3.4 (2.7–4.3)	
Middle	12.3 (11.1–13.7)		4.5 (3.7–5.4)	
Richer	15.5 (13.7–17.4)		8.7 (7.4–10.1)	
Richest	17.7 (15.5–20.2)		13.1 (11.1–15.3)	
**Place of residence**				
Urban	20.5 (18.4–22.8)	*p < 0.01*	13.6 (11.8–15.6)	*p < 0.01*
Rural	43.2 (40.5–46.0)		18.0 (15.9–20.2)	
**Region of residence**				
Barishal	3.4 (2.6–4.3)	*p < 0.05*	1.5 (1.0–2.2)	*p < 0.05*
Chattogram	14.4 (12.4–16.7)		6.3 (5.1–7.8)	
Dhaka	22.7 (20.8–24.7)		12.0 (10.3–14.1)	
Khulna	5.9 (5.1–6.8)		3.3 (2.6–4.2)	
Rajshahi	6.4 (5.5–7.5)		2.9 (2.3–3.8)	
Rangpur	6.1 (5.1–7.2)		3.5 (2.8–4.4)	
Sylhet	4.9 (3.7–6.5)		2.0 (1.4–3.0)	
**Community-level literacy**		*p < 0.01*		*p < 0.01*
Low (≤25%)	7.0 (5.3–9.2)		1.7 (1.2–2.5)	
Moderate (26–50%)	30.1 (26.7–33.9)		14.4 (12.3–16.7)	
High (> 50%)	26.6 (22.7–31.0)		15.5 (12.8–18.5)	
**Community-level poverty**^**+**^				
High	5. 3 (3.4–7.3)	*p < 0.01*	2.7 (1.7–4.3)	*p < 0.01*
Moderate	16.8 (13.8–20.3)		7.3 (5.9–9.1)	
Low	22.6 (19.6–25.8)		8.4 (6.9–10.3)	
Middle-to-richest	19.4 (16.4–22.8)		13.2 (10.9–15.9)	
**Community-level at least four skilled antenatal care visits**				
High (> 50%)	39.1 (36.7–41.6)	*p < 0.01*	16.9 (15.1–18.9)	*p < 0.01*
Low (≤50%)	24.7 (22.5–27.1)		14.7 (12.0–17.9)	

Note: ^+^Women living in a community consisting of only women in the top three wealth quintiles were categorized as living in a “middle-to-richest” community; all other women were categorized on the basis of the proportion of household in their community in the lowest two wealth quintiles: low (25%), moderate (26–50%) and high (> 50%)

Of the four models run separately for at least one skilled ANC visit and at least four skilled ANC visits, the models convergence (*R*_*c*_) were compared. We also compared the AIC (Akaike information criterion), BIC (Bayesian information criterion), and ICC (Intra-class correlation) values to select the best model. The preferred model is the one which has the smaller model convergence (*R*_*c*_), AIC, BIC and ICC value (Table 3; Supplementary Tables 1 and 2). According to these markers, Model 4 (which included individual-, household-, and community-level factors) fitted the data better for each of the outcomes. The ICC values for the null model (Model 1) suggested that around 32 and 72% differences of using at least one skilled ANC visit and at least four skilled ANC visits, respectively, across clusters included in this study. These values were reduced to 1.70e-17 and 60% for at least one skilled ANC visit and at least four skilled ANC visits, respectively, once individual-, household-, and community-level factors were adjusted for in the final model (Supplementary Tables 1 and 2; Table 3).

**Table 3:** Multilevel modelling with Bayesian informative approach to determine the associated with at least one skilled antenatal care uptake and four or more skilled antenatal care uptake in Bangladesh for the year 2014 (using 2014 BDHS)

	At least one skilled antenatal care uptake, aOR (95% Cred. I)	Four or more skilled antenatal care uptake, aOR (95% Cred. I)
Most recent pregnancy: wanted (ref)		
Mistimed pregnancy	0.59 (0.53–0.65)	0.73 (0.66–0.81)
Unwanted pregnancy	0.67 (0.61–0.74)	0.69 (0.64–0.75)
Women’s education: illiterate (ref)		
Primary	1.66 (1.39–1.94)	0.97 (0.91–1.03)
Secondary	1.88 (1.61–2.12)	1.21 (1.10–1.33)
Higher	1.25 (1.11–1.41)	1.85 (1.71–2.00)
Husband’s education: illiterate (ref)		
Primary	1.00 (0.91–1.09)	1.42 (1.29–1.55)
Secondary	1.25 (1.12–1.41)	1.60 (1.48–1.73)
Higher	1.19 (1.06–1.33)	1.62 (1.50–1.76)
Total number of children ever born: ≤2 (ref)		
> 2	1.04 (0.91–1.22)	0.79 (0.71–0.86)
Age at last birth given: ≤ 19 (ref)		
20–34	1.20 (1.07–1.31)	0.91 (0.84–0.96)
≥ 35	1.58 (1.41–1.76)	1.09 (0.98–1.21)
Quality of antenatal care	0.9 (0.08–0.10)	0.40 (0.38–0.41)
Husband’s occupation: agricultural worker (ref)		
Physical labourer	1.06 (0.98–1.16)	1.61 (1.49–1.74)
Services	1.74 (1.49–2.01)	2.52 (2.29–2.76)
Business	2.12 (1.89–2.37)	1.54 (1.40–1.73)
Others	2.17 (1.89–2.45)	2.03 (1.73–2.32)
Exposure to mass media: not exposed (ref)		
Moderately exposed	0.91 (0.79–1.04)	1.09 (1.00–1.18)
Highly exposed	0.92 (0.82–1.01)	1.21 (1.15–1.27)
Wealth status: poorest (ref)		
Poorer	3.31 (2.80–3.81)	1.44 (1.27–1.65)
Middle	1.85 (1.64–2.07)	1.52 (1.43–1.60)
Richer	1.71 (1.48–1.98)	1.53 (1.38–1.69)
Richest	1.83 (1.59–2.07)	1.78 (1.69–1.87)
Place of residence: urban (ref)		
Rural	0.61 (0.56–0.67)	0.67 (0.62–0.72)
Region of residence: Barishal (ref)		
Chattogram	3.46 (3.09–3.87)	0.90 (0.82–0.98)
Dhaka	2.50 (2.12–2.93)	0.98 (0.92–1.05)
Khulna	2.69 (2.29–3.14)	1.20 (1.11–1.31)
Rajshahi	4.20 (3.61–4.82)	1.15 (0.98–1.33)
Rangpur	3.65 (3.12–4.20)	1.10 (1.02–1.18)
Sylhet	3.00 (2.75–3.27)	1.15 (1.08–1.23)
Community-level literacy: low (≤25%, ref)		
Moderate (25–50%)	0.86 (0.75–0.99)	1.37 (1.28–1.47)
High (> 50%)	1.04 (0.92–1.17)	1.42 (1.28–1.59)
Community-level poverty^+^: high (> 50%, ref)		
Moderate (25–50%)	1.24 (1.07–1.42)	0.94 (0.85–1.03)
Low (< 25%)	1.41 (1.22–1.63)	0.97 (0.88–1.08)
Middle-to- richest	0.67 (0.61–0.75)	0.86 (0.78–0.95)
Community-level at least four skilled antenatal care visits (> 50%)		
Low (< 50%)	0.78 (0.70–0.87)	0.19 (0.18–0.0.20)
Constant	2.81 (2.50–3.14)	0.36 (0.33–0.39)
Intra-class correlation (ICC, cluster level)	1.70e-17	0.60
AIC	3455.26	3455.25
BIC	3689.52	3689.52

*Note*: *Ref* reference group, *aOR* Adjusted Odds Ratio, *Cred. I* Credible Interval, estimates were based on informative Bayesian approach with multilevel logistic regression modelling. An informative prior distribution for the regression coefficients was constructed by first fitting separate Bayesian multilevel logistic regression models with non-informative priors (mean = 0, variance = 10,000) to each of the 2004, 2007 and 2011 BDHSs; AIC, Akaike Information Criterion; BIC, Bayesian Information Criterion; ICC, Intra-class correlation. ^+^Women living in a community consisting of only women in the top three wealth quintiles were categorized as living in a “middle-to-richest” community; all other women were categorized on the basis of the proportion of household in their community in the lowest two wealth quintiles: low (25%), moderate (26–50%) and high (> 50%). To see the results for each model, see supplementary Tables 1 and 2

Odds ratios of both outcome variables produced in the final model (Model 4) are presented in Table 3, whereas all model results are presented in Supplementary Tables 1 and 2. A 27% (95% Cred. I, 0.66–0.81) and a 31% (95% Cred. I, 0.64–0.75) lower odds of attending at least four skilled ANC visits were found among women who had a mistimed and an unwanted pregnancy, respectively, compared to women who had a wanted pregnancy. These likelihoods were even lower for at least one skilled ANC visit among women who had a mistimed pregnancy (aOR, 0.59, 95% Cred. I, 0.53–0.65) and women who had an unwanted pregnancy (aOR, 0.67, 95% Cred. I, 0.61–0.74) than women who had a wanted pregnancy.

A number of confounders that were adjusted for were also found to be preventive of having at least one skilled ANC visit and at least four skilled ANC visits. These were women and their husbands who had a lower education, higher number of births, lower quality of available ANC, less exposure to mass media, and lower household wealth. Higher likelihoods of having at least one skilled ANC visit were found among women aged 20–34 years and ≥ 35 years than their counterparts aged ≤19 years. However, the likelihood of having four or more skilled ANC visits was found to be lower among women aged 20–34 years.

Lower likelihoods of ANC uptake were found among women who lived rurally and women who resided in Chattogram region compared to women who lived in an urban area and women who resided in Barishal region. Higher likelihoods of having at least one skilled ANC visit as well as four or more skilled ANC visits were found among women who resided in Khulna, Rangpur, and Sylhet regions. Women who resided in communities with moderate and higher literacy were reported to have around a 40% higher uptake of four or more skilled ANC visits than women residing in a community with low literacy. Lower likelihoods uptake of ANC visit (both at least one and at least four) were also found among women residing in the middle-to- richest communities than their counterparts residing in communities with higher poverty. Compared to the higher community-level uptake of four or more skilled ANC visits (at least 50% of community people), lower community-level uptake of at least four skilled ANC visits (< 50 of community people) was also found to be significant to lowered odds of having four or more skilled ANC visits.

## Discussion

The primary aim of this study was to provide empirical evidence for the association between pregnancy intention and subsequent ANC uptake using nationally representative household survey data from Bangladesh. Around one-quarter of all pregnancies that ended in live births within 3 years preceding the survey were unintended. There were around 27 and 31% lower likelihoods of having the recommended minimum four skilled ANC visits among women who had a mistimed pregnancy and women who had an unwanted pregnancy, respectively, compared to women who had a wanted pregnancy. Likelihoods were even lower for at least one skilled ANC visit, with women who had a mistimed pregnancy and women who had an unwanted pregnancy reporting 41 and 33% lower odds of having at least one skilled ANC visit, respectively, than women who had a wanted pregnancy. This indicates the risk of increasing adverse consequences (e.g. maternal and child deaths) associated with no or lower ANC uptake. Policies are required for the early detection of women who have unintended pregnancies through family planning workers and adding them to mainstream healthcare services.

The SDGs call for universal health coverage and the reduction of preventable maternal and under-five deaths in LMICs [12]. However, the provision of health coverage does not necessarily translate into actual uptake. Our study indicates that unintended pregnancy (at around 26%) is a barrier to ANC uptake, and therefore, will challenge Bangladesh’s ability to achieve its SDG targets. This finding is in line with previous studies in other LMICs, including Tanzania, Zimbabwe, and Brazil [29–31], although the actual prevalence of unintended pregnancy in Bangladesh is lower than other LMICs [22, 44, 45]. However, previous studies in Bangladesh reported no effects of a mistimed pregnancy [9] or an unintended pregnancy [8] on ANC uptake, compared to a wanted pregnancy.

The factors associated with lower likelihoods of ANC uptake following an unintended pregnancy are complex, making designing interventions challenging. A variety of factors related to the pregnancy, the woman and her partner, and psychological responses following pregnancy could be linked with lower likelihoods of ANC uptake [46]. For instance, a mistimed or an unwanted pregnancy are most often identified later than a wanted pregnancy [47], and delay in deciding whether to terminate the pregnancy [48] could result in less time to receive ANC. The characteristics of women and their partners (i.e. already having the desired number of children, and/or lower education and socio-economic status) who report higher unintended pregnancies could also reduce ANC uptake [49, 50]. This link could be due to women’s tendency to rely on experience gained from previous pregnancies, financial difficulties, lower awareness of ANC, autonomy to receive ANC, or some combination of these factors [21]. Moreover, women who have an unintended pregnancy may have psychological problems (e.g. depression, anxiety) that arise from less favourable behaviours, either from their partners, their family members, or both [46]. These are most often in addition to women’s concern about adverse life events (e.g. problems related to work, education, and/or finances), which could prevent them from taking up ANC [51].

In Bangladesh, ANC services are available only in the pre-specified healthcare facilities [13], whose number is usually lower than the actual number of available healthcare facilities [52]. Previous assessments in LMICs found that availability of ANC only in the pre-specified healthcare facilities could reduce the overall uptake of ANC [18, 53, 54]. Given there is no health insurance coverage in Bangladesh, women have to depend mostly on their partners or family members to meet healthcare costs. Moreover, religious and socio-cultural customs in Bangladesh mostly restrict women’s movements during pregnancy. This increases women’s dependency on their partners and/or family members to accompany them to healthcare centres. Together, this contributes to lower uptake of ANC following an unintended pregnancy. The underlying causes are women’s lower autonomy, and their partners/family members unfavourable behaviours as well as unwillingness or inability to expense travel money [46, 51]. These limitations are further amplified for women residing in rural areas and communities with higher illiteracy and poverty, where previous studies have found a higher occurrence of unintended pregnancy [49, 50]. This is because rural areas have a limited number of healthcare facilities, services provided there are deemed lower in quality, and rural women face strong religious and/or cultural opposition to visiting healthcare facilities, particularly when the healthcare facility is far away [55–57]. Under these circumstances, early detection of women’s pregnancy intention and provision of services at the community level are recommended to increase ANC uptake in Bangladesh [15].

Healthcare policies in Bangladesh have primarily been focused on providing free ANC for all women and on promoting media campaigns to substantially increase ANC uptake [58, 59]. However, these policies might be ineffective for women with an unintended pregnancy, as they are most often disadvantaged, among whom this study, as well as other studies in Bangladesh, have found higher odds of not utilizing ANC [8, 9]. This, therefore, leads to multifaceted risk factors of not using ANC among women with an unintended pregnancy. For instance, disadvantageous socio-demographic and societal factors (which are mostly unchanged by the pregnancy experience) are often additional to the healthcare system-level factors and characteristics following the occurrence of an unintended pregnancy. This suggests a need for additional interventions.

Motivating women to use healthcare services could be an effective way to increase ANC uptake among women who have an unintended pregnancy [21]. However, this might not be possible in Bangladesh with the current two-level structure of the maternal healthcare system: maternal healthcare services (provided in clinics/healthcare centres/hospitals by the Directorate General of Health Services [DGHS]) and family planning services (provided at the household level by the Directorate General of Family Planning Services [DGFS]). Therefore, such institution-based maternal healthcare services might not be effective to ensure ANC services use among women who do not visit healthcare centres or who do not continue visiting for ANC. A link between family planning workers and ANC providers in pre-specified healthcare centres for ANC services should be developed to ensure that doorstep ANC is provided to the women who are not motivated to use ANC (because of distance, unintended pregnancy, or any other cause) [60]. This initiative needs a country-level approach to extend the role of maternal healthcare service providers to provide household-level services instead of the current structure of institutional-level services. Additional initiatives are also needed to upskill the current family planning service providers (in total 219,000 providers [56,000 in government sectors and 163,000 in non-government sectors]) to extend their roles to detecting women’s pregnancy intention and ensuring maternal healthcare services use during each pregnancy [59, 61, 62]. Focus is also needed to alleviate the current shortage of family planning service providers (800,000) to ensure they are present in each community, particularly in disadvantaged groups (in terms of ANC uptake and occurrence of unintended pregnancy) [18, 21, 63].

The existing healthcare system in Bangladesh does not focus on religious or cultural norms, which are often barriers to ANC uptake, particularly in communities with higher illiteracy and poverty and where the occurrence of unintended pregnancy is higher [21, 64]. Importantly, these religious and cultural norms are often more influential than individual-level factors (e.g. poor autonomy, lower education), indicating current individual-level awareness programs on maternal healthcare services might not ensure universal uptake of ANC, which is a target in the SDGs. Therefore, at the community-level, community and religious leaders could be engaged to motivate women and increase awareness of ANC visits. Moreover, it is also important to decentralize healthcare centres, which are currently mainly situated in urban areas, to rural areas and resource-poor settings [13]. Furthermore, new healthcare centres are needed, particularly in Chattogram division, where uptake of ANC is usually found to be lower than other divisions in Bangladesh [8, 20]. This is particularly important as illiteracy and poverty are higher in these settings, which increases unintended pregnancies and reduces ANC uptake [65]. The establishment of 18,000 community health clinics in the sub-district level through a recently completed project (mostly in rural and resource-poor settings) made substantial progress in meeting this target [66]. However, additional policies are needed to ensure the provision of necessary physical and human infrastructure, logistics, and supplies, as well as policies to encourage women (particularly women at a higher risk of not receiving ANC) to receive ANC, as existing healthcare centres have frequently been reported to be low-performing or underutilized [64].

The current study has a number of strengths. A major strength is that it analysed a nationally representative sample through informative Bayesian multilevel logistic regression models, which is considered a proper framework for healthcare services research. In addition to the appropriate adjustment of confounders, we calculated their informative priors from the earlier rounds of BDHSs (conducted in 2004, 2007, and 2011), which were identical (in terms of sampling and response rates) to BDHS 2014 that data analysed. This provides evidence on the validity of informative priors used, and therefore, odds estimated in this study are considered more precise and robust than the classical models. However, responses on pregnancy intention and ANC uptake were collected after delivery, hence could be subject to recall bias. Moreover, since the data were only collected from women who had live births, underreporting might have occurred because of pregnancy termination and the reporting of ambivalent responses on pregnancy intention. In addition, the association between women’s pregnancy intention and subsequent use of ANC were adjusted for available individual-, household-, and community-level factors. The very low value of ICC for at least one skilled ANC visit (1.70e-17) suggested that confounders included can almost fully explain the cluster-level variation of attendance of at least one skilled ANC visit. However, a higher ICC value for four or more skilled ANC visits (0.60) suggested a need to adjust additional factors, including availability and accessibility of healthcare services and motivational factors. However, these factors were not included in the analysis because of the lack of data. Additionally, this study analysed data of the 2014 BDHS, which may be considered dated; however, no other nationally representative survey was conducted after this survey and no major changes to maternal healthcare polices or programs have occurred since this survey was conducted. Therefore, the results can still provide a benchmark for monitoring and evaluating current maternal healthcare services in Bangladesh.

## Conclusions

The findings demonstrate that around two-thirds of women who gave birth in the 3 years preceding the survey received ANC at least once. However, four or more skilled ANC visits, as recommended by the WHO, was just 32% of women. These percentages of ANC uptake varied significantly across women’s pregnancy intention at the time of conception. After adjustment for confounders, we found that having a mistimed and having an unwanted pregnancy were associated with lower odds of ANC uptake compared to having a wanted pregnancy. This suggests that unintended pregnancy (at around 26%) challenges Bangladesh’s ability to achieve its SDG targets of universal health coverage and of reducing preventable maternal and under-five deaths. Early detection of women’s pregnancy intention and initiatives to include women in mainstream healthcare services are therefore important. This can be done by extending the role of family planning providers to detecting pregnancy intention and motivating women to receive ANC, as well as making them collaborate with ANC providers to pre-specified healthcare centres for ANC services. Policies and programs focusing on religious and cultural norms are also important to successfully increasing ANC uptake, particularly among communities where illiteracy and poverty are higher.

## Supplementary information

**Additional file 1: Supplementary Table 1.** Multilevel modelling with Bayesian informative approach to determine the associated with at least one skilled antenatal care visit in Bangladesh for the year 2014 (using 2014 BDHS). **Supplementary Table 2.** Multilevel modelling with Bayesian informative approach to determine the associated with at least four skilled antenatal care visits in Bangladesh for the year 2014 (using 2014 BDHS).

## Data Availability

The data that support the findings of this study are available from the DHS Program, but restrictions apply to the availability of these data, which were used under license for the current study, and so are not publicly available. Data are, however, available from the authors upon reasonable request and with permission of the DHS Program.

## Abbreviations

ANC: Antenatal care
AIC: Akaike Information Criteria
BDHS: Bangladesh Demographic and Health Survey
BIC: Bayesian Information Criteria
ICC: Intra-class correlation
LMICs: Low- and Middle-Income Countries
MDGs: Millennium Development Goals
OR: Odds Ratios
95% Cred I: 95% Credible Interval
SDGs: Sustainable Development Goals
WHO: World Health Organization
