# Supplementary material for: Effect of unintended pregnancy on skilled antenatal care uptake in Bangladesh: analysis of national survey data
Source: Arch Public Health. 2020 Sep 16;78:81. doi: 10.1186/s13690-020-00468-1 (PMC7493902; doi:10.1186/s13690-020-00468-1)
Supplement: Supplementary file 1 — Additional file 1: Supplementary Table 1. Multilevel modelling with Bayesian informative approach to determine the associated with at least one skilled antenatal care visit in Bangladesh for the year 2014 (using 2014 BDHS). Supplementary Table 2. Multilevel modelling with Bayesian informative approach to determine the associated with at least four skilled antenatal care visits in Bangladesh for the year 2014 (using 2014 BDHS). [file 13690_2020_468_MOESM1_ESM.docx]

**Supplementary Table 1:** Multilevel modelling with Bayesian informative approach to determine the associated with at least one skilled antenatal care visit in Bangladesh for the year 2014 (using 2014 BDHS)

|  | Null model | Individual and household-level model (OR, 95% Cred I) | Community-level model (OR, 95% Cred I) | Individual, household, and community level model  (OR, 95% Cred I) |
| --- | --- | --- | --- | --- |
| Most recent pregnancy: wanted (ref) |  |  |  |  |
| Mistimed pregnancy |  | 0.65 (0.58-0.75) |  | 0.59 (0.53-0.65) |
| Unwanted pregnancy |  | 0.93 (0.82-1.07) |  | 0.67 (0.61-0.74) |
| Women’s education: illiterate (ref) |  |  |  |  |
| Primary |  | 2.36 (2.05-2.76) |  | 1.66 (1.39-1.94) |
| Secondary |  | 2.31 (1.98-2.74) |  | 1.88 (1.61-2.12) |
| Higher |  | 1.93 (1.61-2.32) |  | 1.25 (1.11-1.41) |
| Husband’s education: illiterate (ref) |  |  |  |  |
| Primary |  | 0.84 (0.73-0.96) |  | 1.00 (0.91-1.09) |
| Secondary |  | 1.00 (0.83-1.16) |  | 1.25 (1.12-1.41) |
| Higher |  | 1.32 (1.15-1.50) |  | 1.19 (1.06-1.33) |
| Total number of children ever born: ≤2 (ref) |  |  |  |  |
| >2 |  | 0.91 (0.80-1.04) |  | 1.04 (0.91-1.22) |
| Age at last birth given: ≤ 19 (ref) |  |  |  |  |
| 20-34 |  | 1.08 (0.98-1.20) |  | 1.20 (1.07-1.31) |
| ≥35 |  | 1.35 (1.18-1.54) |  | 1.58 (1.41-1.76) |
| Quality of antenatal care |  | 0.10 (0.09-0.11) |  | 0.9 (0.08-0.10) |
| Husband’s occupation: agricultural worker (ref) |  |  |  |  |
| Physical labourer |  | 1.10 (1.01-1.21) |  | 1.06 (0.98-1.16) |
| Service worker |  | 1.80 (1.35-2.36) |  | 1.74 (1.49-2.01) |
| Business person |  | 1.50 (1.33-1.69) |  | 2.12 (1.89-2.37) |
| Others |  | 1.78 (1.56-2.08) |  | 2.17 (1.89-2.45) |
| Exposure to mass media: not exposed (ref) |  |  |  |  |
| Moderately exposed |  | 0.97 (0.85-1.10) |  | 0.91 (0.79-1.04) |
| Highly exposed |  | 0.99 (0.86-1.14) |  | 0.92 (0.82-1.01) |
| Wealth status: poorest (ref) |  |  |  |  |
| Poorer |  | 2.41 (2.00-2.89) |  | 3.31 (2.80-3.81) |
| Middle |  | 2.54 (2.18-3.01) |  | 1.85 (1.64-2.07) |
| Richer |  | 2.04 (1.61-2.33) |  | 1.71 (1.48-1.98) |
| Richest |  | 1.92 (1.61-2.33) |  | 1.83 (1.59-2.07) |
| Place of residence: urban (ref) |  |  |  |  |
| Rural |  |  | 0.68 (0.44-1.03) | 0.61 (0.56-0.67) |
| Region of residence: Barishal (ref) |  |  |  |  |
| Chattogram |  |  | 1.03 (0.68-1.55) | 3.46 (3.09-3.87) |
| Dhaka |  |  | 1.20 (0.78-1.86) | 2.50 (2.12-2.93) |
| Khulna |  |  | 1.12 (0.73-1.74) | 2.69 (2.29-3.14) |
| Rajshahi |  |  | 1.03 (0.67-1.61) | 4.20 (3.61-4.82) |
| Rangpur |  |  | 1.21 (0.76-1.93) | 3.65 (3.12-4.20) |
| Sylhet |  |  | 1.30 (0.79-2.13) | 3.00 (2.75-3.27) |
| Community-level literacy: low (≤25%, ref) |  |  |  |  |
| Moderate (25-50%) |  |  | 0.79 (0.58-1.08) | 0.86 (0.75-0.99) |
| High (>50%) |  |  | 0.41 (0.18-0.95) | 1.04 (0.92-1.17) |
| Community-level poverty^+^: high (>50%, ref) |  |  |  |  |
| Moderate (25-50%) |  |  | 1.22 (0.87-1.69) | 1.24 (1.07-1.42) |
| Low (<25%) |  |  | 1.36 (0.82-2.25) | 1.41 (1.22-1.63) |
| Middle-to-richest |  |  | 1.73 (0.97-3.11) | 0.67 (0.61-0.75) |
| Community-level at least four skilled antenatal care visits (>50%) |  |  |  |  |
| Low (<50%) |  |  | 0.12 (0.02-0.77) | 0.78 (0.70-0.87) |
| Constant |  | 3.28 (2.86-3.73) | 1.94 (0.91-4.17) | 2.81 (2.50-3.14) |
| Intra-class correlation (ICC, cluster level) | 0.32 | 0.19 | 0.10 | 1.70e-17 |
| AIC | 5817.35 | 3766.46 | 3971.86 | 3455.26 |
| BIC | 5836.58 | 3920.58 | 4070.56 | 3689.52 |

Note: Ref; reference group; aOR, Adjusted Odds Ratio; Cred. I, Credible Interval, estimates were based on informative Bayesian approach with multilevel logistic regression modelling. An informative prior distribution for the regression coefficients was constructed by first fitting separate Bayesian multilevel logistic regression models with non-informative priors (mean=0, variance=10,000) to each of the 2004, 2007 and 2011 BDHSs; AIC, Akaike Information Criterion; BIC, Bayesian Information Criterion; ICC, Intra-class correlation. ^+^Women living in a community consisting of only women in the top three wealth quintiles were categorized as living in a “middle-to-richest” community; all other women were categorized on the basis of the proportion of household in their community in the lowest two wealth quintiles: low (25%), moderate (26-50%) and high (>50%).

**Supplementary Table 2:** Multilevel modelling with Bayesian informative approach to determine the associated with at least four skilled antenatal care visits in Bangladesh for the year 2014 (using 2014 BDHS)

|  | Null model | Individual and household-level model (OR, 95% Cred I) | Community-level model (OR, 95% Cred I) | Individual, household, and community level model  (OR, 95% Cred I) |
| --- | --- | --- | --- | --- |
| Most recent pregnancy: wanted (ref) |  |  |  |  |
| Mistimed pregnancy |  | 0.73 (0.64-0.83) |  | 0.73 (0.66-0.81) |
| Unwanted pregnancy |  | 0.64 (0.58-0.69) |  | 0.69 (0.64-0.75) |
| Women’s education: illiterate (ref) |  |  |  |  |
| Primary |  | 0.93 (0.83-1.02) |  | 0.97 (0.91-1.03) |
| Secondary |  | 1.07 (0.95-1.20) |  | 1.21 (1.10-1.33) |
| Higher |  | 1.64 (1.46-1.85) |  | 1.85 (1.71-2.00) |
| Husband’s education: illiterate (ref) |  |  |  |  |
| Primary |  | 1.26 (1.12-1.40) |  | 1.42 (1.29-1.55) |
| Secondary |  | 1.50 (1.35-1.66) |  | 1.60 (1.48-1.73) |
| Higher |  | 1.38 (1.25-1.52) |  | 1.62 (1.50-1.76) |
| Total number of children ever born: ≤2 (ref) |  |  |  |  |
| >2 |  | 0.78 (0.69-0.86) |  | 0.79 (0.71-0.86) |
| Age at last birth given: ≤ 19 (ref) |  |  |  |  |
| 20-34 |  | 0.83 (0.78-0.88) |  | 0.91 (0.84-0.96) |
| ≥35 |  | 0.97 (0.84-1.11) |  | 1.09 (0.98-1.21) |
| Quality of antenatal care |  | 0.42 (0.39-0.44) |  | 0.40 (0.38-0.41) |
| Husband’s occupation: agricultural worker (ref) |  |  |  |  |
| Physical labourer |  | 1.35 (1.24-1.46) |  | 1.61 (1.49-1.74) |
| Service worker |  | 2.89 (2.57-3.22) |  | 2.52 (2.29-2.76) |
| Business person |  | 1.45 (1.29-1.63) |  | 1.54 (1.40-1.73) |
| Others |  | 1.59 (1.38-1.84) |  | 2.03 (1.73-2.32) |
| Exposure to mass media: not exposed (ref) |  |  |  |  |
| Moderately exposed |  | 1.04 (0.93-1.17) |  | 1.09 (1.00-1.18) |
| Highly exposed |  | 1.09 (0.99-1.19) |  | 1.21 (1.15-1.27) |
| Wealth status: poorest (ref) |  |  |  |  |
| Poorer |  | 0.91 (0.80-1.03) |  | 1.44 (1.27-1.65) |
| Middle |  | 0.73 (0.63-0.86) |  | 1.52 (1.43-1.60) |
| Richer |  | 0.90 (0.83-0.99) |  | 1.53 (1.38-1.69) |
| Richest |  | 1.28 (1.07-1.52) |  | 1.78 (1.69-1.87) |
| Place of residence: urban (ref) |  |  |  |  |
| Rural |  |  | 0.68 (0.44-1.03) | 0.67 (0.62-0.72) |
| Region of residence: Barishal (ref) |  |  |  |  |
| Chattogram |  |  | 1.03 (0.68-1.54) | 0.90 (0.82-0.98) |
| Dhaka |  |  | 1.20 (0.78-1.86) | 0.98 (0.92-1.05) |
| Khulna |  |  | 1.12 (0.73-1.74) | 1.20 (1.11-1.31) |
| Rajshahi |  |  | 1.03 (0.67-1.61) | 1.15 (0.98-1.33) |
| Rangpur |  |  | 1.21 (0.76-1.93) | 1.10 (1.02-1.18) |
| Sylhet |  |  | 1.30 (0.79-2.13) | 1.15 (1.08-1.23) |
| Community-level literacy: low (≤25%, ref) |  |  |  |  |
| Moderate (25-50%) |  |  | 0.79 (0.58-1.08) | 1.37 (1.28-1.47) |
| High (>50%) |  |  | 0.41 (0.17-0.94) | 1.42 (1.28-1.59) |
| Community-level poverty^+^: high (>50%, ref) |  |  |  |  |
| Moderate (25-50%) |  |  | 1.22 (0.87-1.69) | 0.94 (0.85-1.03) |
| Low (<25%) |  |  | 1.36 (0.82-2.25) | 0.97 (0.88-1.08) |
| Middle-to-richest |  |  | 1.73 (0.97-3.11) | 0.86 (0.78-0.95) |
| Community-level at least four skilled antenatal care visits (>50%) |  |  |  |  |
| Low (<50%) |  |  | 0.12 (0.02-0.77) | 0.19 (0.18-0.0.20) |
| Intra-class correlation (ICC, cluster level) | 0.72 | 0.68 | 0.64 | 0.60 |
| AIC | 4514.46 | 3766.46 | 3515.23 | 3455.25 |
| BIC | 4532.97 | 3920.58 | 3715.25 | 3689.52 |

Note: Ref; reference group; aOR, Adjusted Odds Ratio; Cred. I, Credible Interval, estimates were based on informative Bayesian approach with multilevel logistic regression modelling. An informative prior distribution for the regression coefficients was constructed by first fitting separate Bayesian multilevel logistic regression models with non-informative priors (mean=0, variance=10,000) to each of the 2004, 2007 and 2011 BDHSs; AIC, Akaike Information Criterion; BIC, Bayesian Information Criterion; ICC, Intra-class correlation. ^+^Women living in a community consisting of only women in the top three wealth quintiles were categorized as living in a “middle-to-richest” community; all other women were categorized on the basis of the proportion of household in their community in the lowest two wealth quintiles: low (25%), moderate (26-50%) and high (>50%).
